# A new factor predicting excessive femoral anteversion in patients with recurrent patellar dislocation

**DOI:** 10.1186/s13018-022-03259-2

**Published:** 2022-07-28

**Authors:** Conglei Dong, Kuo Hao, Chao Zhao, Fei Wang

**Affiliations:** Department of Orthopaedic Surgery, Third Hospital of Hebei Medical University, Ziqiang Road 139, Shijiazhuang, 050051 Hebei China

**Keywords:** Patellar dislocation, Patellar instability, Femoral anteversion, Derotational femoral osteotomy, Medial condylar angle

## Abstract

**Purpose:**

Determining a new imaging method on full-leg standing lower limb radiographs to predict excessive femoral anteversion in patients with patellar dislocation.

**Methods:**

This study included 119 patients with patellar dislocation from January 2014 to January 2021. The femoral anteversion and tibial torsion were measured by CT scanning. The medial condylar angle was measured by the full-leg standing lower limb radiographs. Pearson correlation coefficient was used to evaluate the correlation between rotation parameters and medial condylar angle.

**Results:**

Included patients were divided into DFO group and control group according to whether they received derotational femoral osteotomy (DFO) operation or not. DFO group had significantly higher femoral anteversion (29.8° ± 7.2° vs 23.1° ± 6.5°, *P* < 0.05), higher tibial torsion (28.6° ± 6.9° vs 24.7° ± 7.9°, *P* < 0.05), lower medial condylar angle (154.8° ± 4.7° vs 157.5° ± 6.7°, *P* < 0.05) than control group. Correlation analysis showed that the values of femoral anteversion were significantly correlated with medial condylar angle (r = -0.719, *P* < 0.001).

**Conclusion:**

This study showed that medial condylar angle had a negative correlation with excessive femoral anteversion on the full-leg standing lower limb radiographs. The medial condylar angle can be a good predictor of femoral anteversion and can be used to guide the performance of DFO to treat patellar dislocation in clinical practice.

## Introduction

Patellar dislocation is a common condition, which is often seen in children and adolescents [1]. Many factors are related to patellar dislocation, which includes anatomical factors, such as trochlear dysplasia, patellar tilt, patella alta, increased Q angle (due to femoral anteversion and tibial torsion), and generalized ligament relaxation [2–4]. Biomechanics has proved that femoral anteversion is an independent risk factor of patellar dislocation [5]. Excessive femoral anteversion can lead to abnormal patellofemoral load and the tendency of lateral subluxation [6]. It is also a risk factor of medial patellofemoral ligament reconstruction (MPFLR) failure [7–11]. Therefore, derotational femoral osteotomy (DFO) has been considered as a better option for patellar dislocation with excessive femoral anteversion, and good clinical results have been achieved during follow-up [12].

The values of femoral anteversion is traditionally measured by computed tomography (CT) or magnetic resonance imaging (MRI) of hip, knee, and ankle joints, which extend from the anterosuperior iliac spine to toes [13]. Although there are many different measurement methods, the standard measurement of femoral anteversion angle in a healthy adult population is highly dependent on the identified markers and imaging technology used, which may lead to differences between different measurement results [14–17]. In addition, the risks of exposure to excessive and unexpected radiation and the high costs cannot be ignored [17]. Therefore, determining a new imaging method to predict excessive femoral anteversion may be beneficial for surgical treatment of patellar dislocation.

It is still unknown whether the morphology of the distal femoral condyle is related to femoral anteversion. To show this relationship, this study introduced the concept of medial condylar angle on the full-leg standing lower limb radiographs, which is a new predictive value to predict excessive femoral anteversion. Full-leg standing lower limb radiographs are the simplest and most direct imaging method for patients with patellar dislocation, which is convenient to obtain both in outpatient and ward work [18].

Therefore, this study was performed assessing the predictive role of medial condylar angle on femoral anteversion in patients with patellar dislocation on the full-leg standing lower limb radiographs. This study hypothesized that excessive femoral anteversion may lead to the decreased medial condylar angle in patients with patellar dislocation and medial condylar angle may be a guiding factor to instruct the performance of DFO to treat patellar dislocation.

## Materials and methods

### Patients selection

This study was approved by our Institutional Review Board, and informed written consent was obtained from all patients. The patients with patellar dislocation from January 2014 to January 2021 were analyzed retrospectively. The inclusion criteria were as follows: (1) patients who developed patellar dislocation at least once or patients with clinical and imaging diagnosis of patellar dislocation which needed surgical treatment; (2) patients with mature bones; (3) patients who had CT scans and full-leg standing lower limb radiographs within one week before the operation; (4) the interval between CT and full-leg standing lower limb radiography was within one weeks. Patients were excluded if they had traumatic dislocation, previous knee surgery, previous fractures involved knees or no appropriate imaging examination. The surgical protocol was determined by the surgeon in accordance with the medical history, physical examination, and imaging data. Among the patients who met the inclusion criteria, the patients who received DFO operation were classified as the DFO group, otherwise the control group.

### Image evaluation

CT scans and full-leg standing lower limb radiographs were performed within one week before the operation as a part of preoperative planning. A CT scanner (Siemens Somatom Perspective, Germany) was used to obtain CT scans ranging form the anterosuperior iliac spine to the toes with patients in a supine position with their knees fully extended and feet facing upward. The field of view (FOV) was ranging from 220 to 450 mm, according to the size of the patients. Other parameters were as follows: tube voltage, 130kVp; Tube current, 110-140mAs; Slice thickness, 0.5 mm; Matrix, 512 × 512 pixels. The full-leg standing lower limb radiographs were taken when patients stood straight with both knees fully extended and feet facing forward, and evenly distribute their body weight between both limbs.

All measurement data were collected from the Picture Archiving and Communication System (PACS) workstation and measured by one experienced researchers blindly and randomly at the same time (CD). The mean value measured by two researchers was the final data (CD and KH). If there were abnormal values, another independent researcher would re-measure them (FW). To determine the reliability of inter-observer and intra-observer measurements, the intra-class correlation values (ICC) were calculated.

### Femoral anteversion

**Fig. 1:**
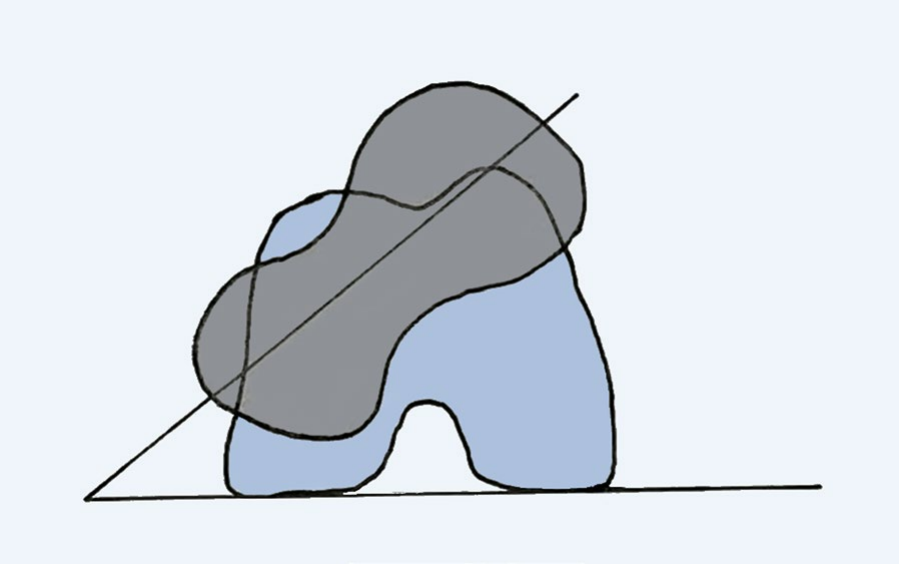
The measurement of femoral anteversion

The femoral anteversion was defined as the angle between the posterior condyle line and the projection of the femoral neck axis [19]. The posterior condyle line was the line passing through the posterior point of the medial anf lateral femoral condyle [19, 20]. The femoral neck axis was the line connecting the center of the femoral head and the center of the femoral neck base [20]. The positive angle indicated femoral anteversion and the negative angle referrd to femoral retroversion (Fig. 1).

### Tibial torsion

**Fig. 2:**
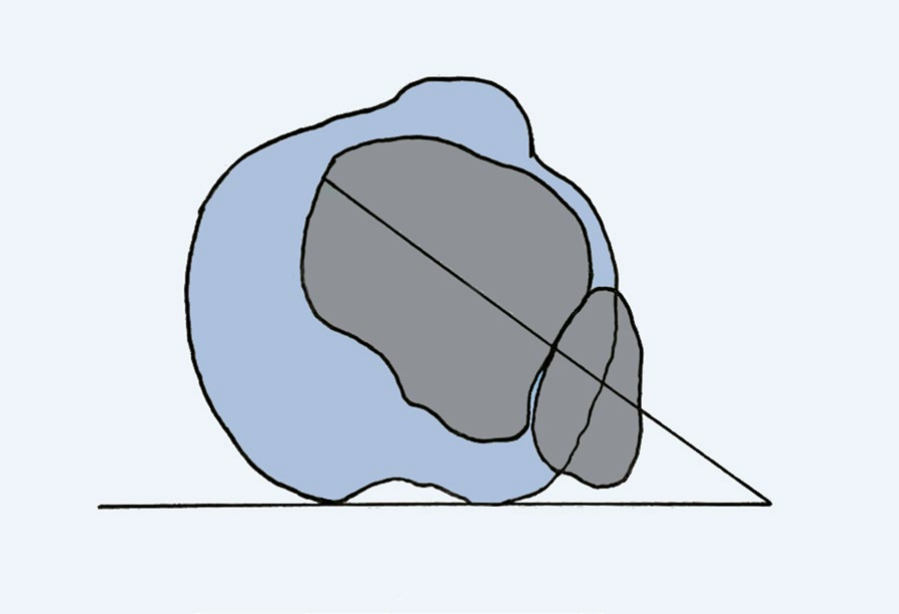
The measurement of tibial torsion

The tibial torsion was defined as the angle between the tangent line of posterior edge of tibial plateau and the intermalleolar axis, which was the line passing through the center of the medial and lateral malleolus [21] (Fig. 2).

### Medial condylar angle

**Fig. 3:**
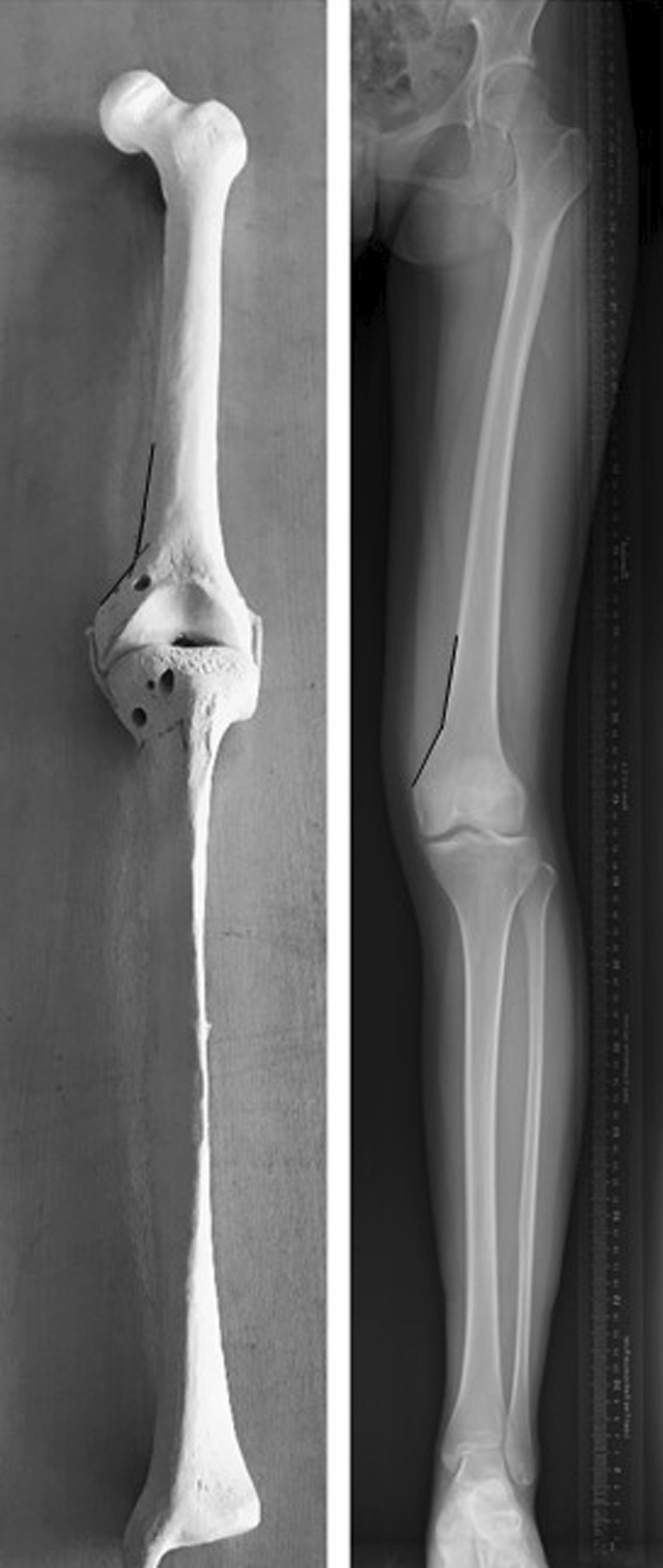
The measurement of medial condylar angle

The medial condylar angle was defined as the angle between a straight line along the surface of the medial femoral condyle and another straight line along the medial distal femur extending to the transition between femoral shaft and medial femoral condyle on the full-leg standing lower limb radiographs (Fig. 3).

### Statistical analysis

Descriptive analyses were presented by mean and standard deviation (SD) for continuous variables and frequencies with percentages for discrete variables. The independent-samples t-test was used to analyze the differences between the DFO group and the control group and Levene's test was used to examine the homogeneity of the data. Pearson correlation coefficient was used to evaluate the correlation between rotation parameters and medial condylar angle. The intra-class correlation coefficient (ICC) was calculated to evaluate the reliability of medial condylar angle. The measurement series of 30 patients (15 from the DFO group and 15 from the control group) were randomly selected and either repeated by 1 researcher at intervals of 2 weeks or independently measured by 2 different researchers. An ICC greater than 0.80 indicated good consistency. All analyese were conducted using SPSS software (version 21.0, SPSS Inc, Armonk, NY). *P* < 0.05 was defined as statistically significant.

## Results

**Fig. 4:**
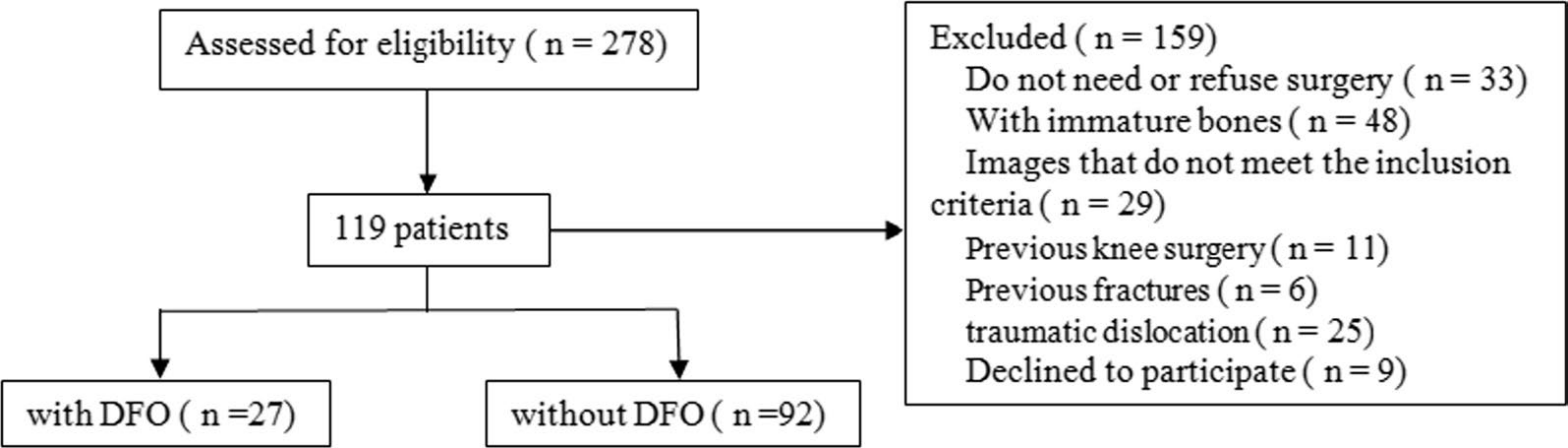
Flowchart of patients screening

278 patients with patellar dislocation hospitalized in our department from January 2014 to January 2021 were selected. According to the inclusion and exclusion criteria, 119 patients consisted of 41 males and 78 females were included in the study, of which 27 underwent DFO and 92 not (Fig. 4). Demographic characteristics are shown in Table 1. No significant difference in sex or age was found between the two groups (*P* > 0.05).

**Table 1:** Demographic characteristics

	DFO group	Control group	*P*
Gender ratio	20 women: 7 men	68 women: 24 men	> 0.05
Age, y	32.9 ± 9.5	31.3 ± 9.9	> 0.05
BMI	22.8 ± 2.5	23.1 ± 3.0	> 0.05
Sides, n	15 left: 12 right	44 left: 48 right	> 0.05

In the DFO group, femoral anteversion was 29.8° ± 7.2°, tibial torsion was 28.6° ± 6.9°, the medial condylar angle was 154.8° ± 4.7°. In the control group, femoral anteversion was 23.1° ± 6.5°, tibial torsion was 24.7° ± 7.9°, the medial condylar angle was 157.5° ± 6.7° (Table 2). The inter-observer and intra-observer ICC were 0.912 and 0.924 for femoral anteversion, 0.899 and 0.914 for tibial torsion, 0.925 and 0.917 for medial condylar angle, respectively. This indicated that the medial condylar angle showed a good inter-observer and intra-observer consistency.

**Table 2:** Descriptive statistics for measurements of lower limb rotational parameters

	DFO group		Control group		*P*
	Mean	SD	Mean	SD	
Femoral anteversion, °	29.8	7.2	23.1	6.5	< 0.05
Tibial torsion, °	28.6	6.9	24.7	7.9	< 0.05
medial condylar angle, °	154.8	4.7	157.5	6.7	< 0.05

*SD* Standard deviation

There were significant statistical differences in femoral anteversion, tibial torsion and medial condylar angle between DFO group and control group (*P* < 0.05). This showed that there was a smaller medial condylar angle in patients undergoing DFO operation. Correlation analysis showed that the values of femoral anteversion were significantly correlated with medial condylar angle (*r* = − 0.719, *P* < 0.001). However, tibial torsion (*r* = 0.058, *P* = 0.334) showed no significant correlation with the medial condylar angle.

## Discussions

The most important finding in this study was to establish a new angle, which can be used to predict excessive femoral anteversion in patients with patellar dislocation. The medial condylar angle was smaller in patients undergiong DFO than control group, with statistical difference. Correlation analysis showed that the values of femoral anteversion were significantly correlated with medial condylar angle.

The femoral anteversion can be measured by MRI or CT. Although these two methods provide reliable measurement, they have some limitations, such as cost, moving artifacts, and measurement complexity. For example, CT scanning may bring disturbing radiation exposure risks, especially for young patients [17]. Botser et al. compared the measurement methods of femoral anteversion between CT and MRI. They reported that compared with MRI, the value measured by CT was larger, with an average difference of 8.9° [13]. However, due to the complex three-dimensional shape of the femur, the measurement of femoral anteversion can not be accurately obtained in the two-dimensional model, which leads to its limited value in a preoperative plan for DFO. Therefore, it is necessary to guide patients with patellar dislocation to undergo DFO by simple full-leg standing lower limb radiographs.

Although many studies measured femoral anteversion in different ways, no studies have evaluated this angle in full-leg standing lower limb radiographs of patients with patellar dislocation according to an easy-to-use imaging parameter [14, 15, 20, 21]. A smaller medial condylar angle means higher possibility of DFO operation in patients with patellar dislocation, because excessive femoral anteversion was associated with smaller medial condylar angle. Therefore, medial condylar angle can be used as an alternative method to screen patients undergoing DFO surgery. The full-leg standing lower limb radiographs may be helpful for surgeons to clinically evaluate and screen patients who may meet the indications of DFO.

Because of the excessive femoral anteversion, the rotation of the femur leads to the morphological changes of distal femur. Due to the natural anatomical features of lower limbs, the position of medial femoral condyle in healthy individuals was in good alignment. Tear of ligament, deformity of bone structure, and other factors that may affect the force vector of the knee joint may lead to change of distal femur [20]. The disorder of internal biomechanical structure may lead to the loss of good rotational alignment of the knee joint. In patients with patellar dislocation, the rotational deformity was one of the factors, which may lead to the disorder of medial femoral condyle. In patients with mild rotational deformity, the internal ligament structure can compensate for the abnormality of bone structure, to avoid abnormal image examination and clinical symptoms.

There are some limitations to this study. Firstly, there are some errors in the measurement method of medial condylar angle. Because the transition between the distal femur and the medial femoral condyle is an arc, the medial condylar angle is only a parameter to preliminarily judge the femoral anteversion. The purpose of this study was to establish a parameter that can be easily visualized on the full-leg standing lower limb radiographs and can be used to evaluate the rotational deformity. Secondly, the parameter proposed in this study does not include all the factors that lead to the patellar dislocation, because only a few landmarks were analyzed. Further research will be conducted to analyze other factors. Third, this study is a case series study without a control group of normal knee joints without patellar instability. Although there are few patients with abnormal medial condylar angle without patellar instability, the control group may be an important assistant.

## Conclusions

This study showed that medial condylar angle had a negative correlation with excessive femoral anteversion on the full-leg standing lower limb radiographs. The medial condylar angle can be a good predictor of femoral anteversion and can be used to guide the performance of DFO to treat patellar dislocation in clinical practice.

## Data Availability

All of the data and materials are available.

## Abbreviations

DFO: Derotational femoral osteotomy
MPFLR: Medial patellofemoral ligament reconstruction
CT: Computed tomography
MRI: Magnetic resonance imaging
ICC: Intra-class correlation coefficient
